# A Developmental Perspective on Young Children’s Understandings of Paired Graphics Conventions From an Analogy Task

**DOI:** 10.3389/fpsyg.2020.02032

**Published:** 2020-08-18

**Authors:** Jean-Michel Boucheix, Richard K. Lowe, Jean-Pierre Thibaut

**Affiliations:** ^1^LEAD-CNRS, UMR 5022, University of Bourgogne Franche-Comté, Dijon, France; ^2^School of Education, Curtin University, Perth, WA, Australia

**Keywords:** graphicacy development, graphic convention understanding, analogy task, children, school-books

## Abstract

The present study investigated children’s understanding development of multiple graphics, here paired conventions commonly used in primary school textbooks. Paired graphics depicting everyday objects familiar to the children were used as the basis for an analogy task that tested their comprehension of five graphics conventions. This task required participants to compare pictures in a base pair in order to complete a target pair by choosing the correct picture from five alternative possibilities. Four groups of children aged 5, 6, 8, and 10 years old respectively (total *N* = 105), completed 45 analogy task items built around nine conceptual domains. Results showed mainly an overall increase of comprehension performance with age for all the tested conventions. There were also differences between the five conventions and an interaction between age and convention type. Further, children’s explanation of the conventions (justification of the choices in the analogy task) were also analyzed. This investigation showed the analogy task answers were a more reliable measure of the “actual” level of understanding of the conventions than the justification themselves. The findings show that younger students tried to actively compare the pictures of the pairs and to search for a relevant meaning of the pairs, however, the youngest children have a limited capacity to interpret paired graphic conventions and our results suggests that this aspect of graphic conventions develops slowly but effectively over the course of children’s schooling. Because “graphicacy” knowledge and skills are not typically taught in primary school classrooms (in contrast with literacy and numeracy), its development is likely acquired incidentally with increasing exposure to varied paired graphics during primary school education. Given the high reliance of today’s educational resources on graphics-based explanations, the results from this study may signal a need for (i) for more attention to learning graphics conventions (and more generally to graphics explanations) from teachers in primary school and (ii) for a better design of the graphics with their contextual accompanying texts and captions, from designers.

## Introduction

In recent years, the proportion of pictorial information in school textbooks (both print materials and e-books) seems to have increased substantially (for example, a study by Bétrancourt et al., 2012, revealed that the great majority of the content of the pages of recent primary school books – 8 to 11 years old- contained multiple graphics, multiple representations, text and pictures, and especially paired graphics, see also Di Sessa, 2004). This increase has been particularly pronounced in STEM areas and encompasses a wide variety of depiction types (such as diagrams, drawings, photos, videos and animations). Research indicates that combinations of pictures with text are far more educationally effective than text alone. This is the well-known multimedia effect that has been supported by a large number of experimental studies (see Moreno and Mayer, 1999; Mayer, 2009, 2014). A recent meta-analysis by Pastore et al. (2017) found an overall positive effect of the multimedia principle on comprehension performances (*r:0.48*).

### From Text and Graphic Comprehension to Graphics Conventions Understanding

**FIGURE 1:**
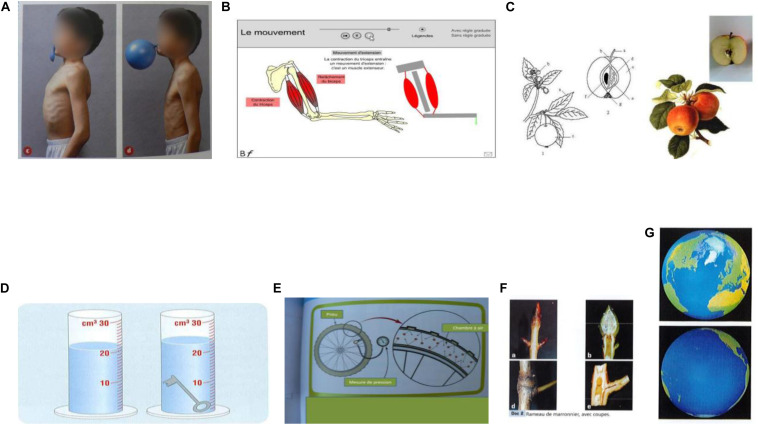
Paired graphics from sciences primary school books, and free science web sites, respectively from left to right: **(A)** before-after from the book Coll. Tavernier, “Sciences expérimentales et technologies,” J. Erb, S. Charpiot, F. Lucas, C. Claveau, Y. Le Ray, p. 76, Bordas Ed., 2003; **(B)** realistic-schematic, animation from “Toutes les Sciences” Cycle 3, digital manual, Nathan Ed, 2010; **(C)** whole-cross section, from Wikipedia web site “apple”; **(D)** before-after process, from the Netherlands science primary school paper book, 2010; **(E)** close-up view, from “Science Aspects 1 “G. Linstead, O. Goyder, G. Przywolnik, L. Salfinger, T. Herbert, p. 223, Sydney: Pearson Heinemann, Eds., 2009; **(F)** whole-cross section, from the book” Sciences” Cycle 3, J.M. Rolando, G. Simonin, P. Pommier, J. Nomblot, J.F. Laslaz, S. Combaluzier, p. 50, Magnard Ed., 2003; **(G)** different views of the same object from “A nous le Monde,” Cycle 3, SEDRAP, P. Beyria & al., CNED, G. Bée & al., p. 133, SEDRAP ed., 2001.

Despite the positive findings mentioned above, it is clear that different types of depictions are not equally effective in promoting learning. According to Levin and Mayer (1993), the most common graphics in documents such as school textbooks were “*decorative and/or representational,”* with only a small percentage of explanatory graphics (see also, Levin et al., 1987; Levin, 1989; Gyselinck, 1996). However, Sung and Mayer (2012) found that “instructive graphics,” (i.e., those that are both explanatory and directly relevant to the instructional goal of their accompanying text) were significantly more effective than graphics that were appealing or decorative but not instructionally relevant. In much of the previous multimedia-oriented research on learning from text and graphics, priority was given to how effectively the text-based information had been processed. This dominant focus is present even in studies that address the referential connections and integration between these two forms of representation (Schmidt-Weigand et al., 2010; Leopold et al., 2015; Schüler, 2017; Désiron et al., 2018; and more recently Schnotz and Wagner, 2018; Zhao et al., 2019). In contrast, very few investigations have been primarily focused on the processing of graphics on their own right (see Schmidt-Weigand et al., 2010). More than 20 years after seminal studies on learning from text and graphics by Levin et al. (1987) and Levin (1989) two recent exploratory studies indicated that (i) the use of multimedia information in science and technology textbooks was far more prevalent than in earlier years, and (ii) the number and variety of explanative graphics used was far greater than reported by Levin and Mayer (1993). These two studies prompted fundamental questions about potential challenges faced by primary school children in order to process such graphics effectively. The first study (Bétrancourt et al., 2012) surveyed the type and nature of graphics found in school textbooks targeting 10–11 years old children (Grade 4 and 5, i.e., late primary school). It examined the use of graphics in a range of widely used science/technology textbooks from different countries (Australia, France, Netherlands, Switzerland, and United Kingdom). As would be expected, the depictions were highly varied. However, in contrast with previous findings (Levin and Mayer, 1993), most of them were explanatory rather than decorative. A notable feature of the textbooks examined in the 2012 survey was the prevalence of multiple rather than single graphics. In most cases, these consisted of a pair of graphics which indicates that this simplest combination could be considered as a multiple graphic *prototype*). These paired graphics were used for a wide range of purposes, including showing related realistic and abstract depictions, portraying ‘before and after’ states, and presenting different views of the same stimulus (Figure 1). Although there was considerable variation in the types of content represented by the paired graphics, the same finite set of generic conventions was used repeatedly. Further, the survey by Bétrancourt et al. (2012), showed also that graphics were included in contexts, e.g., accompanied with texts of different length, some very short, other longer, in such way that graphics came with not only expository texts, but captions, labels and references. However, often, the content of these texts was not explicitly connected and related to the graphics and/or did not provide precise explanations which enable or help the graphics processing: in sum there was a lack of text-picture “coherence” principle (Mayer, 2014). Then, often, textbooks gave no explicit instruction about how children should interpret these conventions or the types of processing activities that they should undertake in order to use paired graphics effectively as a tool for learning. Rather, it seems to be assumed that children would already be equipped to handle these requirements. Of course, teachers may provide scaffolding which eventually acculturates learners into interpreting graphics in a particular way. However, scaffolding opportunities are not systematized, and textbooks are also widely used out of the school time. Finally, a scientific approach of graphics comprehension involves a distinction between text and pictures investigations.

More fundamentally, there are several basic skills that children must possess in order to benefit from paired explanatory graphics. They must understand that the component pictures are related and therefore should be *compared* (rather than treated independently): regularities regarding spatial proximity between pictures and order of the pictures might help. This comparison involves both *within* picture and *between* picture processes. The types of comparative processing required depends on the specific depictive convention that is instantiated in a particular paired graphic (for example, a graphic pair that involves the realistic/abstract convention presents an information set that is very different from the set of information presented by a graphic pair involving the before/after convention – see Figure 1). Therefore, in order to process a graphic pair as intended, children must have sufficient knowledge of these different conventions and be able to invoke and then to apply the appropriate convention successfully.

The second study (Boucheix et al., 2013a) involved 21 children (11 years old) and 18 adult students (20 years old). It investigated the comprehension (measured via verbal responses) of 37 paired graphics taken from the Grade 5 primary school science textbooks referred to above that were presented to participants one at a time. The data indicated that while the great majority of the paired graphics were easily understood by all adult participants (more than 75%), substantially fewer (59.6 %) were understood by the children for whom the textbooks were intended. It appeared that part of the reason for this difference could have been that the children did not always understand the conventions used in the paired graphics. Further, eye movement data obtained from the participants showed that while adults’ inspections tended to be concentrated on the relevant areas of both graphics of each pair (rather than on irrelevant areas), children tended to fixate relevant and irrelevant information equally. However, the preliminary nature of this study did not allow a distinction to be made between (a) the effect of specific knowledge related to paired-graphic conventions, and (b) the effect of prior knowledge about the topics depicted in the graphics. Further, there were limitations in the verbal protocol-self report approach used for data collection. In particular, it was sometimes difficult to determine exactly what the child participants meant by their verbalizations because of ambiguities and explanatory inadequacies. The present paper builds on the two exploratory studies referred to above by using a more rigorous methodology and better controlled materials to pursue the issue of children’s understanding of paired graphics. For the purpose of this study, we conceptualized these graphics as consisting of two different but related pictures placed adjacently that are intended to be interpreted together. The goal of the present study was therefore to examine early development in the comprehension of conventions commonly used in paired graphics.

In order to process a paired graphics’ convention effectively, children need to (1) understand that both pictures represent an object (or action), (2) recognize the objects, situations, and/or processes that are depicted in both images (3) recognize that the two graphics represent different instantiations of the same situation (4) understand the *abstract nature* of the relation between the two depicted objects (or actions). For example, understanding a pair that displays a conventional viewpoint and a longitudinal cross-section of the same object requires a correct identification of the object in the cross-section view but also, more deeply, understanding that the cross-section view is a special point of view on the object, that is grasping the relation between the two views. This requires a correct mapping of the elements seen in the object’s classical representation (conventional viewpoint) and the elements provided by the cross-section.

### Paired Graphics and the Early Development of Pictorial Competence

At first sight, pictures could be regarded as intrinsically effective representations that pose none of the challenges for learners long associated with text-only resources (Mayer and Sims, 1994). However, this view seems simplistic. For example, the fact that a young child can recognize a photograph of his or her own house does not mean that he/she would be able to interpret an abstract architectural plan of the same building. Such sophisticated technical graphic representations can only be understood if the viewer possesses the relevant specialist technical knowledge and skills. Their interpretation relies on the viewer’s ability to decode the highly specialized depictive conventions that these graphics use to present their referent subject matter. As with other methods of symbolic representation, there are three key aspects involved in understanding graphic conventions: (i) a realization that there is the *intention* to refer to something else, (ii) an appreciation that the representation is in a *stand-for* relation to the referent, (iii) an understanding of the way the representation refers to its referent (DeLoache et al., 2003; Deloache, 2004; Tare et al., 2010; Uttal and Yuan, 2014). The ontogenesis of symbol understanding has been the subject of numerous studies. For example, 9-month olds often try to grasp photographs as if they were the real objects, whereas 18-month olds do not (DeLoache et al., 2003). Further, 3 years old understand scale models, whereas many 2.5 years old fail to do so (DeLoache, 1995). It has also been shown that even though young children understand that symbols are objects in their own right and representations of other entities (the dual-representation hypothesis, Deloache, 2000), this understanding remains fragile, especially when superficial similarity between the model and the referent is not perfect (DeLoache et al., 1991; Chiong and DeLoache, 2013).

It seems that designers of the symbolic graphic displays that are so widely used today may attribute an unrealistically high level of transparency to the meaning of such representations, especially for children (Hiniker et al., 2016). However, it is becoming apparent that younger children may lack the skills required to grasp the designer’s intended meaning, something that is potentially highly problematic in an educational context that increasingly relies on explanatory graphics. More generally, the ability to understand and interpret graphics has received little attention in educational research to date, despite having been an “implicit” aspect of many other studies with very diverse goals (Balchin, 1976, 1985; Wainer, 1980; Matthews, 1986; Milsom, 1987; Boardman, 1990; Hadjidemetriou and Williams, 2002; Anning, 2003; Cox et al., 2004; Postigo and Pozo, 2004; Roth et al., 2005; Ainsworth, 2006; Hegarty et al., 2009; Lowrie et al., 2011).

### Processing of Paired Graphics

#### Comparison Processes

Boucheix et al. (2013a) revealed that the processing of paired graphics (as also multiple graphics) during comprehension involved substantial *comparisons* of the two depictions. This result accords with the broader findings from cognitive psychology and conceptual development, that comparison activities are central to learning (e.g., Gentner, 2010). The importance of such comparisons has been noted across a wide variety of different fields such as category learning (Andrews et al., 2011; Augier and Thibaut, 2013), schema acquisition (Gick and Paterson, 1992), conceptual change (Gadgil et al., 2012), and categorization of perceptual stimuli (Kok et al., 2013). In the specific case of between-picture comparisons, the type of content presented in each of the pictures being compared can have crucial effects on learning outcomes. This is exemplified by Kok et al. (2013) in which adult participants’ comparisons of paired graphics (chest X-ray images) were used to study their learning of radiological indicators of diseases in medical diagnosis. One group of medical students compared radiographs of diseases with radiographs from normal patients while the other medical student group studied only radiographs of diseases (pairs of disease images). On a visual diagnosis test, students who compared disease with normal images during study were better able to diagnose focal diseases than students who had studied disease images only More broadly, most studies contrasting comparison conditions with no-comparison conditions suggest that comparisons lead to deeper conceptual understanding and better generalization. Indeed, no-comparison situations may lead to superficial perceptually based generalizations (for example, an apple to a ball) whereas comparison situations contribute to the discovery of unifying non-salient properties such as taxonomic commonalities (e.g., two objects belong to the same category of furniture) or non-salient perceptual properties (e.g., object textures) that tend not be noticed if participants see an object in isolation (e.g., Thibaut, 1991; Gentner and Namy, 1999; Gentner and Gunn, 2001; Namy and Gentner, 2002; Augier and Thibaut, 2013; Thibaut and Witt, 2015). Gentner and colleagues describes the learning mechanism as starting with surface features, leading to the progressive discovery of deeper similarities between images. Features within one picture are progressively matched with features in the other picture (Gentner and Markman, 1997). The more similar the two pictures (or the more they share perceptual features), the easier it is to discriminate the relevant features or extract key relations.

The matching processes involved in comparison activities that are beneficial for learning may also need to be considered in the development of the ability to comprehend paired graphics conventions. However, to investigate this possibility, it is important that the graphics to be compared are age-appropriate, especially in terms of processing (executive functions) costs (Richland et al., 2006; Augier and Thibaut, 2013). In this respect, young children are capable of dealing with tasks involving comparisons. However, as shown by Augier and Thibaut (2013) even though younger children (4-years old) were able to benefit from comparisons, providing more relevant information did not benefit them, by contrast with 6 years old.

#### Progressive Learning of Paired Graphics Conventions?

During their schooling, children are repeatedly exposed to various paired graphics conventions. This exposure occurs across a range of distinct content domains (science, technology, history, geography, etc.) and for different types of subject matter within those domains. The paired graphics that embody these conventions are often accompanied by explanatory texts and further pictures that assist in their interpretation. Children encounter many and varied examples of such use of paired graphics across the course of their primary education. Further, as a result of this exposure, students should progressively acquire the capacity to make increasingly fine grain discriminations between different paired graphics conventions and their specific meanings. For example, they may first consider similar a specific convention (say, a whole/cross-section paired graphic of an orange) with a more general convention (say, a before/after pair showing the orange with a knife before it was cut and afterward). This interpretation is not intrinsically wrong, however, by the end of primary school, such interpretation would no longer be expected because of children’s far greater experience with these conventions. For these older children, whole/cross-section should have become a more specific convention with a precise and possibly more abstract meaning that is distinguished from the more generally applicable before/after convention.

#### Paired Graphics and Conceptual Development

General conceptual development may also play a role in the comprehension of paired graphics. In particular, because certain paired graphics conventions involve changes in object position from one picture to the other (such as side-view/top-view), the development of spatial abilities may influence some aspects of their comprehension. For example, understanding a paired graphic that shows both side and top views of an object may require the learner to perform a mental rotation. Frick et al. (2013) showed that mental rotation abilities are beginning to develop between the ages of 3 and 5 years. Thus, it could be expected that paired graphics conventions involving substantial changes in viewing position or object orientation would be understood later than a paired graphic convention such as the whole view/close-up view convention which does not involve such change.

Conceptual development may also influence generalization, abstraction and transfer abilities. The ability to generalize and transfer a paired graphics convention from the more frequent and prototypical exemplar of the convention to a different, less frequent and semantically more distant exemplar of the same convention would be expected to increase with age. For example, in school text books (Bétrancourt et al., 2012), the whole/cross-section convention is very frequently used for living entities such as fruit, plants and animals. In these cases, the function of this convention is to show the inside components and structure of the organism that are usually invisible from the outside. The ability to generalize the whole/cross-section convention from such prototypical examples to a far broader range of instances, and less likely, (such as non-living objects or structures, like the cross-section of a hat or of a bottle for example) is likely to increase with age. In sum, the semantic distance between the prototypical exemplar of a given convention and a more unfamiliar exemplar of the same convention is likely to have an effect on the comprehension performance of this given convention (see also Table 1, section “Materials and Methods”).

**TABLE 1:** Definition-criteria used for used convention design.

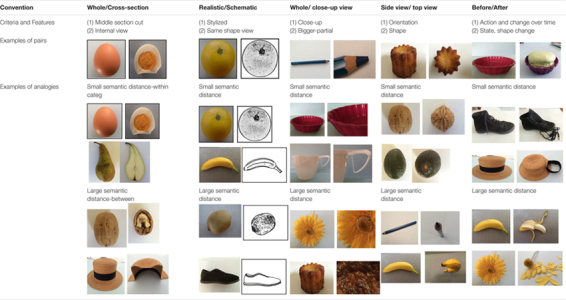

### Paired Graphic Convention Comprehension Assessment in Children

**FIGURE 2:**
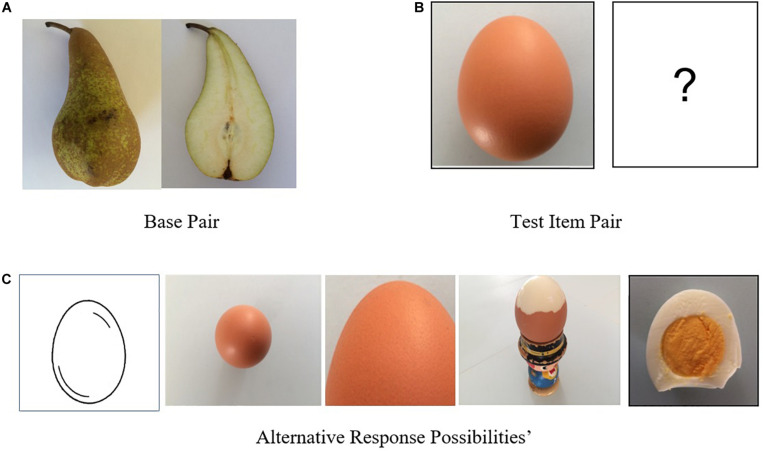
Example item for a cross section analogy. Here the cross section convention is first presented with a paired graphic that uses an peer as **(A)** the base pair subject matter (top left). The participants’ task was to find the correct answer for the egg (top right), **(B)** the test Item Pair, by choosing from the five displayed **(C)**. Alternatives Responses Possibilities (second row) and placing the chosen picture in the empty rectangle (correct answer is rightmost picture).

In their preliminary study, Boucheix et al. (2013a) had used self-report and verbal protocols to investigate comprehension of paired graphics. Although such approaches are effective for adult participants, they could be relatively ineffective in terms of judging children’s comprehension or knowledge of the stimulus materials. For young children especially, verbal justifications are likely to be un-reliable, particularly when they require complex syntactic structures (e.g., expressing causal or complex temporal structures) (Clark, 2009). Children’s verbal justifications might also fail when the vocabulary necessary to express complex relations is beyond the reach of the children involved. In recent years, many experiments with designs that avoid reliance on children’s production of verbal information have been used by developmental psychologists. In many cases, these methods often based on induction and/or generalization like the one we use in the present study, have revealed much earlier competences than methods based on verbalization (see Gelman, 2003, for example). These more recent investigations show advantages in using direct behavioral measures involving tasks that are better suited to children’s processing abilities than too verbally oriented approaches. In order to avoid the limitation of only relying on verbal explanations from young children, the present research recruited a well-established analogy task to provide a more age-appropriate measure of the comprehension of relationships. Analogy tasks have been successfully used in early cognitive development research and in psychometric investigations, in conceptual development, categorization and problem solving studies. Recently, they have been successfully used in pre-linguistic children (Ferry et al., 2015),

The analogy task used in the present study was of the form ‘A is to B as C is to D,’ (A: B::C:D). This approach involves the comparison of a *base pair* (A and B) and a *target pair* (C and D). Most frequently, adults identify the relation holding between items in the A: B pair, then, they apply this relation to the *target pair* pictures or words (see Richland et al., 2006; Holyoak, 2012; Hofstadter and Sander, 2013). Many previous studies showed that by the time children reach 3 or 4 years of age, they are able to use this type of analogy task with familiar stimuli and/or with proper training (e.g., Goswami and Brown, 1990; Richland et al., 2006; Christie and Gentner, 2010; Thibaut et al., 2010a). Further, analogy tasks are typically designed, by definition, *to be an index of relation extraction* which is central in the symbolic representations we consider here. Indeed, children who understand the conventions targeted in present study would be able to identify the abstract relation holding in the base pair (e.g., the second stimulus is a cross-section of the first object) and apply it to the second pair. To ensure that children’s selection reflected their understanding of the convention, the options included in the alternatives set were depictions of the object shown in picture C that embodied other non-target conventions. For example, in Figure 2, below in the Method section, the base pair (A–B) is a whole pear and the sagittal cross section view of a pear, while C is an egg. The target object is then to be chosen from the set of possibilities displayed in the second row that are also views of the egg corresponding to the five conventions studied in this research. This was done to prevent alternatives being discarded by participants on conceptual basis that would be unrelated to the conventions being studied here. This is the approach found in most analogy-based studies (see Christie and Gentner, 2010; Thibaut et al., 2010a, b).

### Paired Graphic Convention Comprehension Assessment in Children

In the present study, the paired graphics reasoning analogy task described above was used to investigate the extent to which children from different age groups understand five graphics conventions that are commonly used in textbooks and e-books: whole/cross-section, whole/close-up, before/after, realistic/schematic, and side-view/top-view.

From consideration of the theoretical concepts and issues discussed in the previous section, the following set of hypotheses were developed:

*Hypothesis 1*. Older participants were predicted to have higher scores on the analogy test (*H1a*) and be more likely to generate appropriate justifications than younger participants (*H1b*).

*Hypothesis 2.* Differences in the comprehension scores were predicted to occur across the five conventions used in this study. This hypothesis is based on the contention that these convention types would differ in the level of processing demands they imposed on the participants. For example, conventions that resulted in a high level of perceptual similarity between the graphics in a pair and preservation of visuospatial structure (e.g., the realistic/abstract convention) should be understood at a younger age than conventions that resulted in substantial perceptual and structural change (e.g., whole/cross section, before/after, and side/top-view) (c.f., Gentner and Smith, 2013). As noted earlier, understanding a paired graphic that involves two very different viewpoints on an object likely requires the viewer to perform a mental rotation. Frick et al. (2013) showed that mental rotation abilities are only just beginning to develop between the ages of 3 and 5 years. Thus, it could be expected that paired graphics conventions involving substantial changes in viewing position or object orientation would be understood at an older age than a paired graphic convention that does not involve such change.

*Hypothesis 3.* For errors, it was predicted that the type of chosen distractor would vary across ages. We hypothesized that choices based on perceptual features only would decrease with age level”

## Materials and Methods

### Participants

Participants were 105 children (52 female) from French primary schools. To ensure that each participant sample was representative of the intended population, the schools were chosen such that varied socio-cultural backgrounds were equally represented in each age group. Children were divided into four age groups according to class level in order to obtain samples with ages of approximately 5, 6, 8 and 10 years old. 17 children (*M* = 5.23 years old, *SD* = 0.44) were included in the 5 years old age group, 32 children (*M* = 6.47 years old, *SD* = 0.51) were include in the 6 years old age group, 18 children were included in the 8 years old age group, and (*M* = 8.7 years old, *SD* = 0.55), and finally, 38 children (*M* = 10.37, *SD* = 0.60) were included in the 10 years old group. These four age groups were chosen in order to provide useful differences in the relative degree to which the children had been exposed to graphic conventions in school (little or no exposure, low exposure, and high exposure).

Concerning participants’ educational background with regard to textbooks, schoolchildren in France typically first encounter textbooks only toward the very end of kindergarten (preschool) when they are 5 to 6 years old. Proper introduction of textbooks does not occur until the 1st year of primary school at age seven. From then, textbook use becomes more regular and increases through the remaining years of primary school (i.e., until 9–10 years old).

However, the degree to which textbooks are used for a particular age cohort is also influenced by the specific learning methods implemented within particular schools and by individual teacher choice. Regarding this last point, definitive research evidence about patterns of variations in textbook use across primary schools is unfortunately lacking.

In the present study, there were differences in the number of children across groups due to the inevitable variations in school classroom size. As well as obtaining parental and teacher consent for participation, teachers were consulted to ensure that none of the children included in the sample had learning disabilities, were color blind or had any developmental issues.

### Experimental Design

A two factor experimental design was used with age group as the between subject factor (four levels) and type of convention the within subjects’ factor (five levels).

### Material Design and Task Organization

The core material for this study was sets of paired graphics depicting a range of different types of familiar subject matter that instantiated the five conventions specified above. As shown in Table 1, the difficulty of items within object categories used in the analogy task was varied. This was done by making some of the tested objects fairly similar and others less similar. Items involving the analogical pairing of similar objects were anticipated to be easier to answer than those where less similar objects were paired. For example, it was expected that it would be easier to correctly identify a cross-section of an orange if the base pair depicted a kiwi fruit than if the base pair depicted a hat. However, our goal in the use of varied categories was to be sure to assess the extent to which graphics convention comprehension processes could generalize. These paired graphics were used as the basis for producing analogical items (A is to B as C is to D) as exemplified in Figure 2.

Table 1 details the five convention categories and provides examples of how they were operationalized in the experimental stimuli.

The second row of Table 1 (*criteria and features*) presents two main defining aspects of each convention: (i) the action employed in order to implement the convention, and (ii) the perceptual consequences of that implementation. For example, in the whole/cross-section convention the action employed is to make a vertical cut through the middle of the object along its long axis. The consequence is that the internal structures of the object then become available to visual perception. Example pairs showing objects before and after the application of the convention are given in the third row of the table. Comparison of the five conventions reveals both commonalities and differences in their defining features. First, most of them are associated with a change in the object’s appearance, orientation or shape. An exception is the realistic/schematic convention where only the graphic treatment of the object is changed. In this case, the two depictions comprising the pair are relatively similar in terms of both their overall perceptual properties and structural characteristics. Such obvious similarities tend not to be present for the other four conventions because of the disruptions caused by manipulations of the objects or viewing regimes that are employed in order to apply those conventions. The different conventions can be further distinguished in terms of the particular set of distinctive changes they involve. For example, application of the whole/close-up convention results in a change in the object’s appearance and shape, but no change in its orientation. In contrast, orientation change is the defining feature of the side-top view convention. Such variations are likely to have consequences for how these different conventions need to be processed by the viewer in order to interpret them appropriately. For example, cognitive processing of the side/top-view convention might require the ability to mentally simulate the spatial rotation of the object from the side to the top view. Such mental rotation ability could be more difficult for younger than for older children (see hypotheses above).

The common before/after convention deserves special attention because it appears to be very different in nature from the other conventions. In particular, it seems to be more difficult to characterize with a similar degree of precision because it involves *any* type of action applied to an object that subsequently results in *any* type of change in that object. Hence, both the cause and effect are very open (essentially undefined). In some cases, the change over time may be relatively small so that the overall structural characteristics of the object in the two pictures remain very similar. This is exemplified in Figure 1A, where the fundamental body structure of the child remains much the same (with only minor changes in its form). In this case, it is relatively easy for a viewer who compares and contrasts the material in the two depictions to notice the key relevant features that have changed between the ‘before’ and ‘after’ pictures. However, in other cases the change between the two pictures can be far more dramatic, as illustrated in Table 1 by the examples in the final cell of the Before/After column:

• The intact banana (picture 1) versus the peeled banana together with its peelings (picture 2), or
• The intact flower (picture 1) versus the flower from which all the petals have been removed and placed next to the stem (picture 2).

In both these examples, pictures 1 and 2 of each pair could be considered as the same object modified, and not as two different identical objects.

The fourth row of Table 1 provides examples of analogies based on each type of convention in which differences in semantic (conceptual) distance between base pair and target pair are involved. For each convention, two types of items were devised - within category and between category items. To illustrate this distinction, we will consider the cross-section convention. When the base pair represents the cross-section of an orange and the target pair a cross-section of an apple, the semantic distance was small since both pairs come from the same category, fruits (*within category* items). However, when the base pair involves the cross-section of an egg and the target pair the cross-section of a shoe, the semantic distance between the two pairs was larger because they belong to different object categories (*between category* items). Further, a cross-section of a shoe is highly unlikely, and un-ecological (relatively to the school textbooks contents, Bétrancourt et al., 2012; Boucheix et al., 2013a), however, such graphics exemplars were designed to try to assess experimentally the level of generalization of the interpretation of the convention. The stimulus materials used in the present investigation consisted of approximately the same proportion of within and between category items for each of the five conventions.

The previously discussed differences in the characteristics of the conventions suggest that the processing demands they impose on children may vary. For conventions that are more difficult to process, it could be expected that interpretative competence would develop later than for those with lower processing demands. For example, because of the perceptual-structural similarity between the elements of the pair, the realistic-schematic convention was expected to be easier for children to process than the other conventions (such as the whole-cross section). In contrast, the side-top view convention was expected to be one of the most difficult because this convention likely requires the ability to mentally rotate an object. On this analysis, the capacity to deal effectively with the side/top view convention should develop later than the realistic-schematic convention (see hypotheses above).

Regarding the analogy task, if the relation holding between pictures A and B in a graphic pair is understood, it should allow the participant to apply this relation to picture C in order to find appropriate picture D amongst a set of potential responses. Finding of the correct answer was thus assumed to indicate that the child understood the targeted graphic convention. The comprehension performance score in this study was based on the total percentage of correct answers for the analogy task across all five conventions. Immediately after giving each answer, children gave a verbal justification for their response. These verbalizations were classified and analyzed according to the basis of the justifications involved (as detailed below.

Each analogy item was presented individually on a large touch screen (Wacom 21) using software specially designed for the experiment. The five alternatives displayed in the second row were presented in a random order to avoid location (rank) repetition and possible spatial strategy learning. The software automatically recorded the nature and latency (in milliseconds) of the response for each item. The base paired graphics used in this experiment as the stimuli for the analogy task were high definition photographs of nine familiar everyday objects: an orange, a banana, a kiwi fruit, a flower, an egg, a cup, a hat, a shoe, and a cake mold. Participants’ familiarity with each of the objects was checked to avoid any potential prior knowledge effect. The size and rendering of the photographs (or their modified versions) were tested to ensure that each provided a clear and appropriate depiction of all relevant aspects of the subject matter. Further, the set of images comprising each of the analogy items was examined, and pre-tested in a pilot study, to eliminate any potential ambiguities with respect to which convention was being targeted by that item.

With nine objects and five conventions for each, the main experimental material provided a total of 45 individual analogy items of the type shown in Figure 2. Two additional training analogies were used to ensure participant familiarity with the task requirements. These analogies used another very simple convention (whole object/the same object in pieces) that was not one of the conventions being investigated in this study. For each item, the child was asked to use a finger to touch the chosen picture (which when touched moved immediately to the empty frame and replaced the ‘?’). After the training phase, the 45 experimental analogy items were presented in a random order. Children were also asked to provide a verbal justification for their choice of each item (“Please tell me why you chose this picture?”), with these justifications being recorded.

### Procedure

The investigation took place in a quiet room at the participating schools with each child taking part individually. The analogy task instructions were based on those used for previous studies in our lab, and that had been validated with younger children. They were as follows: *“Notice that these two pictures go well together (experimenter pointing to pictures A and B). Your task is to find among these pictures (experimenter pointing across the second row) which one goes with C (experimenter pointing to C) in the same way that A goes with B. When you have found the picture, touch it with your finger and the picture will automatically go to the empty square near the first picture of the two (experimenter pointing to picture C and space D). If you think you made a mistake, you can correct it by touching another picture. Each time you will have to explain to me why you choose that picture.”* If a participant changed a selection after an initial response was given, justification was always requested once the final response had been provided. Success on the two training items indicated that participants had good comprehension of the task requirements. Following the training trials (with additional task explanation given if needed to ensure that the instructions were well understood), the participant completed the 45 analogy test items and provided a justification for the choice made after each item. The main analogy task began once the child had successfully completed the training items.

Upon completion of all the test items, a further control task was undertaken by each child to check familiarity with the objects in their various pictorial manifestations. In this main control task, each individual base picture of the nine objects and each individual picture of the five corresponding alternative depictions utilizing the conventions was presented to the participant on the screen. The child was asked to name the object shown in the picture in order to check that it was recognized for all depictions, all viewing point, used during the investigation. For example, is an orange presented in cross section format still recognized as an orange? This additional control task ensured that any incorrect responses given in the analogy task were not due to a failure to recognize the object rather than to deficiencies in the capacity to deal with graphic conventions. The duration for the whole session ranged from 30 to 40 min.

### Coding and Analysis

For each convention type, the distribution of the choices made across the possible responses (the target and the four distractors) was calculated and transformed into percentages. Each *answer choice* received a score of 1 when “correct,” e.g., expected, and 0 when “incorrect,” e.g., not expected, thus providing a maximum total score across the five conventions of 45 points. Note that the categories correct and incorrect did not mean that the child answer was right or wrong in term of interpretation, rather, it meant that the child choice was expected, or not expected, relatively to the to the convention tested. A score out of 9 for each convention type was also calculated and these *correct choice scores* transformed into percentages. Further, in order to obtain a developmental profile of the extent to which the different conventions could be distinguished, each error was classified according to the type of convention involved. For each convention, the *Mean response time* in seconds was also determined.

The verbal justifications were coded according to four categories. (i) *Appropriate* when a relevant, fully correct and explicit explanation was given that included at least the first main criterion specified in the second row of Table 1 (e.g., for the cross section convention: “I chose this picture because the object is bisected” or “I chose this picture because we can see half of the orange”); (ii) *Partially Appropriate* when the explanation was relevant but only partly correct and/or indicated implicitly rather than directly, and including only the second categorization criterion given in Table 1 (which was mostly the perceptual consequence of the main criteria, see Table 1, e.g., for cross section: “I chose this picture because we see the inside of the object”); (iii) *Inappropriate* when the explanation was neither relevant nor correct (e.g., for cross section: “I chose this picture because the object is bigger”; generic criteria: “I chose this picture because it is different” (iv) *None* when no justification could be given by the child (or when the child explicitly told to the experimenter: “I don’t know”). Participants answers were scored by two independent raters, with inter-rater agreement, chance corrected Cohen’s kappa, being high 0.97. Justification scores were also calculated. On the basis of this scoring of the answers, Table 5 (see below in the results section) proposes a qualitative categorization of the justifications, which gives a detailed comparison of a series of representative examples in each category of justification across the different age groups. Regarding the naming control task (where appropriate synonyms were considered as correct), the mean percentage of correct answer was calculated.

## Results

Firstly, data from the control naming task (i.e., object recognition) will be presented. This analysis concerned the conditions necessary for legitimate interpretation of the data from the main analogy task investigation. Secondly, the distribution of answers across the five possible choices (correct target versus four incorrect answers) will be reported for each convention type. With regard to Hypothesis 1a, and Hypothesis 2, the mean percentages of correct answers for each age group and each convention will be compared. Then, with regard to Hypothesis 3, the results for distribution of choices across the four distractors will be given. Finally, with regard to Hypothesis 1b, these previous analyses will be followed by an analysis of justifications, and their associated relations and correlations with the correct answer choices. A qualitative description of the justifications types and accuracy, based on the use of the *verbatim* data of each age group for each convention type will be presented before reporting the quantitative analyses of the justification scores and their relations with the correct choice answers scores for the analogy task.

### Objects Naming Task

Almost all the individual pictures used in this study were recognized and correctly named, irrespective of age group. Mean recognition frequencies were 93.4% (*SD* = 8.15), 94.14% (*SD* = *6.02*), 95.93% (*SD* = 4.43) and 96.72% (*SD* = 3.84) for the 5, 6, 8, and 10-years old age groups, respectively. A one factor ANOVA conducted on the mean percentage of pictures of objects named correctly (with age as between subject factor) indicated no significant difference between the age groups, *F*(3,101) = 4.96, *p* = 0.12, ns. Any significant differences that were present between age groups in correct choice scores would therefore not be due to a lack of familiarity with the depicted objects.

### Answer Choice Scores

**FIGURE 3:**
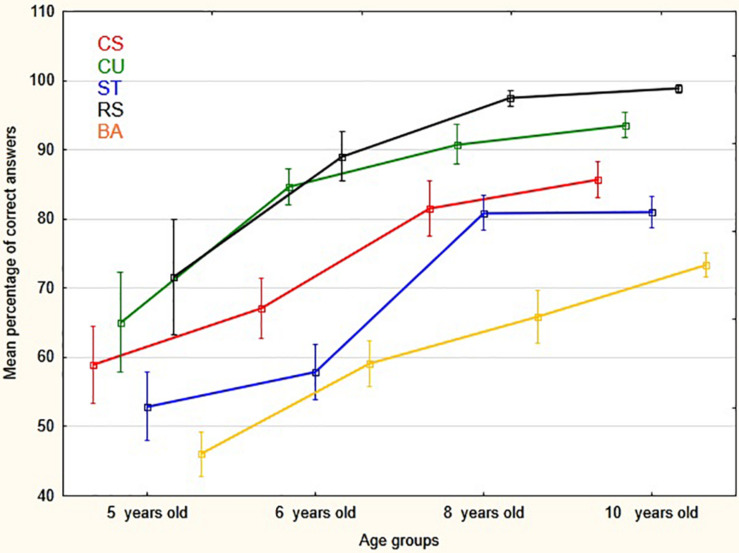
Mean percentage (and vertical bars standard errors) of correct answers by age groups and conventions (CS, Whole-Cross section; CU, Close-Up views; ST, Side-Top views; RS, Realistic- Schematic; BA, Before-After).

Figure 3 and Table 2 show the distributions of the answer choices (expressed as percentages) across the five possible responses.

**TABLE 2:** Ratio, % (and SD) of the distribution of the different possible choices for each convention and each group (the sum of each raw is 100%).

Convention	Choice Age	Whole/Cross- section	Realistic/Schematic	Whole/close up view	Side view/top view	Before/After
Whole/Cross-section	5 y	58.9 (23.10)	3.26 (7.62)	8.10 (11.58)	9.31 (8.38)	20.42 (16.34)
	6 y	67.06 (24.71)	2.17 (6.71)	3.12 (7.59)	6.42 (8.33)	21.57 (15.41)
	8 y	81.48 (17.04)	0.01 (0.05)	1.23 (3.59)	1.85 (4.26)	15.43 (13.81)
	10 y	85.67 (15.69)	0.29 (1.80)	2.04 (6.76)	1.46 (3.80)	10.52 (9.64)
Realistic -Schematic	5 y	1.96 (5.87)	71.65 (34.55)	6.61 (8.88)	11.19 (15.71)	8.57 (15.50)
	6 y	3.17 (6.52)	88.71 (19.68)	2.08 (5.24)	2.56 (7.90)	3.47 (7.70)
	8 y	0.61 (2.61)	97.45 (4.91)	0.69 (2.95)	1.23 (3.59)	0.00 (0.00)
	10 y	0.29 (1.80)	98.83 (3.45)	0.00 (0.00)	0.87 (3.03)	0.00 (0.00)
Close view -up view	5 y	7.92 (10.25)	3.26 (6.53)	65.03 (29.77)	14.46 (15.55)	8.66 (9.51)
	6 y	4.16 (5.46)	2.78 (7.46)	83.98 (14.36)	7.68 (11.10)	1.39 (4.68)
	8 y	3.08 (6.38)	0.01 (0.05)	91.35 (11.44)	3.09 (6.38)	1.85 (4.26)
	10 y	1.74 (4.10)	0.29 (1.80)	93.56 (11.14)	4.38 (7.54)	0.29 (1.80)
Side view -top view	5 y	11.76 (15.45)	7.84 (9.43)	14.38 (12.27)	52.94 (20.23)	13.07 (14.29)
	6 y	9.50 (10.35)	7.68 (11.80)	16.4 (12.67)	57.81 (23.03)	8.59 (9.18)
	8 y	2.47 (4.75)	1.24 (5.23)	14.81 (7.62)	80.86 (10.65)	0.62 (2.61)
	10 y	4.42 (7.13)	0.58 (2.51)	9.62 (12.31)	82.96 (13.59)	1.79 (4.92)
Before -after	5 y	30.84 (15.96)	2.61 (6.24)	6.80 (11.30)	14.34 (10.61)	45.39 (14.04)
	6 y	25.30 (14.57)	5.64 (13.05)	4.61 (8.95)	7.13 (10.28)	56.99 (19.67)
	8 y	21.68 (11.66)	1.85 (5.72)	4.40 (5.68)	6.79 (8.64)	65.89 (16.27)
	10 y	20.83 (10.20)	1.46 (3.81)	1.46 (3.80)	3.80 (5.93)	73.02 (10.62)

#### Correct Answers Scores

For both the overall total percentage of correct answers and for each convention score, two types of statistical analysis were performed. First, conventional MANOVAs and ANOVAs for interval variables, were performed. Second, Table 1 showed that the between groups variances were not equal (which is very common with children of different age groups, with more variance in younger groups). As a consequence, ANOVAs were complemented with non-parametric analyses.

A repeated measure MANOVA analysis of correct answer scores (see Table 2), with age group as the between subject factor and convention type as the within subject factor, showed a significant effect of age on the comprehension of the conventions *F*(3, 101) = 26.79, *p* < 0.00001, ηp^2^ = 0.44. There was also a clear effect of convention type *F*(4,404) = 55.21, *p* < 0.00001, ηp^2^ = 0.35, with some conventions being correctly identified more often than others. In addition, there was no significant interaction between convention and age, *F*(12, 404) = 1.44, *p* = 0.14, ηp^2^ = 0.041. As a consequence, this last finding reflects the main effect of age group for each of the convention type (see Figure 3). Further, the non-parametric, Kruskal–Wallis ANOVA also showed a significant difference between age groups: *H*(3, 105) = 52.56, *p* < 0.00001, mean rank for respectively 5, 6, 8, and 10 years old age groups: 18.85, 38.29, 62.61, 76.10; median test: Chi-Square = 36,50257, *df* = 3, *p* < 0.0001. In sum, hypothesis 1a was supported. In addition, it should be pointed out that response times, that were also recorded for each item during the analogy task time, showed the same trends of performance as the correct answers scores. However, and because no separate hypotheses were made about response times, they were not analyzed further.

### Answer Choices Distribution Analysis

Regarding Hypothesis 3, if choice errors for a particular convention are not equally distributed across the four distractors, this would suggest that choice was preferentially directed toward one of the other conventions. Such selection bias could indicate that the specific meaning features of the tested convention are not yet completely fixed resulting in assimilation between conventions. It seems likely that such assimilations, that are not really wrong, would be higher in the younger children that in the older children, showing a developmental trend. Thus, there could be effects of particular conventions on one another where an age group is more likely to make an unexpected choice of some particular type when viewing a convention of some other particular type. For example, as shown in table one, 5 years old children chose mainly the correct whole/cross section analogy answer for the whole/cross section convention (58.9%). However, 20.42% of them chose the before/after convention instead. This result suggests possible assimilation of the shared general temporal characteristic between the two conventions. The whole/cross section convention could be interpreted as including a temporal aspect: a cross section of an orange may require a first step in which the whole object is cut in a certain way. However, as shown in Table 2, for 10-year-old children, there is a much lower prevalence of such liken of the before/after convention and the whole/cross section convention (10.5%). This is consistent with the hypothesis of the whole/cross section convention having acquired a more restricted and specific meaning which has now a specific feature different from the before/after convention. To address this issue more generally, we conducted analyses of alternative incorrect responses that had been given for each of the conventions. This was done by examining the distribution of distractor incorrect choices for each convention type. Non-parametric Kruskal–Wallis ANOVA for multiple independent sample were performed, with age as the between subject factor and distractor type as the within subject factor (the mean percentage frequency with which each of the 4 different distractor types was chosen). In Table 3 below, the results of the Kruskal–Wallis ANOVAs are presented. For each convention, significant decrease of the choices of detractors are detailed.

**TABLE 3:** Results of the Non-parametric Kruskal–Wallis ANOVA on the effect of age groups on distractors choices for each convention type.

Conventions	Significant decrease in the choice distractors		
	Distractors choices	*H*-values = With H (3,105)	Mean ranks for 5, 6, 8, 10 years old
Whole/Cross-section	Close/Up views	10.88, *p* = 0.012	67.64, 52.85, 48.50, 48.70
	Side/Top views	19.88, *p* = 0.0002	71.05, 60.10, 44.66, 42.94
	Before/After	12.82, *p* = 0.005	60.97, 64.53, 50.47, 40.92
	Realistic/Schematic	No significant	
Realistic/Schematic	Close/Up views	19.66, *p* = 0.0002	68.47, 54.46, 49.50, 46.50
	Side/Top views	16.18, *p* = 0.001	70.23, 51.14, 49.88, 48.32
	Before/After	18.92, *p* = 0.0003	65.50, 57.73, 46.50, 46.50
	Whole/Cross-section	No significant	
Close/Up views	Whole/Cross-section	8.60, *p* = 0.04	65.05, 56.75, 49.94, 45.89
	Side/Top views	11.62, *p* = 0.009	70.38, 55.64, 43.61, 47. 44
	Before/After	25.07, *p* = 0.0001	73.38, 49.92, 53.33, 46.31
	Realistic/Schematic	9.16, *p* = 0.03	60.26, 56.30, 48.00, 49.34
Side/Top views	Whole/Cross-section	9.96, *p* = 0.02	61.08, 61.79, 41.38, 47.48
	Close/Up views	No significant	
	Before/After	28.51, *p* = 0.0001	71.35, 64.46, 38.02, 42.22
	Realistic/Schematic	22.68, *p* = 0.0001	67.44, 62.35, 43.77, 43.02
Before/After	Whole/Cross-section	No significant	
	Close/Up views	No significant	
	Side/Top views	14.96, *p* = 0.002	74.88, 52.06, 52.77, 44.10
	Realistic/Schematic	No significant	

In sum, these results are consistent with hypothesis 3. For incorrect answers, and overall, there are differences between ages in the choice of the type of distractor. First we observed a strong decrease in the mean percentage of distractors choices, especially after 5 years old. Second, for some conventions there was no difference between age group (realistic/abstract convention) because of the small number of incorrect, non-expected, choice for most of the conventions, or on the contrary because there were many assimilations (*conflates*?) between alternative conventions (before/after). Third, for the other conventions (whole/cross section, whole/close-up, top/side view) the trend seems to show progressive specification and restriction of the meaning and use of each convention. The amount of assimilation among convention remained low: For the realistic-schematic convention, the most frequent assimilation with the side/top view convention reached only 11%, and disappeared after 5 years old. For the whole/close-up convention, the most frequent assimilation with the side/top view convention reached only 14%, dropped dramatically and disappeared after 5 years old. For the side-view/top-view convention, assimilation rates seem to remain relatively higher than for the other conventions (see Table 2). This result was similar for the before/after convention, for this latter, assimilation rates remain high, between 31 and 21% across ages, see Table 2.

In addition, in order to address the question of whether a distractor type, and which one, was selected most often for a given convention, independently of the quantitative amount of the choice–e.g., for example, whether before/after is more likely to be selected than the other types for the whole/cross-section convention, as appears to be the trend in Table 2, an analysis of the distribution rank of each of the four distractors, for each convention type, was conducted for each age group. Non-parametric Friedman ANOVAs, for the comparison of multiple dependent variable, were performed on the four distractors as within group factor and for each age group. The results are presented in Table 4.

**TABLE 4:** Results of the Non-parametric Friedman ANOVA on the effect of distractors types on distractor choices for each age group.

Conventions	The four distractors choices ranks differences by age		
	Ages	Friedman ANOVAs Chi. Sqr. (χ^2^) *df* 3 values and significance	Mean ranks distractors orders: CS = Cross-Section; CU = Close-Up; TV = Side-Top; RS = Realistic-Abstract; BA = Before-After
Whole/Cross-section	5 y	**χ^2^** = 15.76, *p* = 0.001	BA: 3.32, TV: 2.56, CU: 2.29, RS: 1.82
	6 y	**χ^2^** = 47.86, *p* < 0.00001	BA: 3.58, TV: 2.45, CU: 2.01, RS: 1.95
	8 y	**χ^2^** = 36.67, *p* < 0.00001	BA: 3.69, TV: 2.22, CU: 2.14, RS: 1.94
	10 y	**χ^2^** = 56.51, *p* < 0.00001	BA: 3.42, CU: 2.27, TV: 2.25, RS: 2.05
Realistic/Schematic	5 y	**χ^2^** = 10.24, *p* < 0.02	TV: 2.91, BA: 2.67, CU: 2.47, CS: 1.94
	6 y	**χ^2^** = 2.07, *p* = 0.56, ns.	BA: 2.60, CS: 2.54, CU: 2.45, TV: 2.39
	8 y	**χ^2^** = 2.00, p. 57, ns.	TV: 2.61, CU: 2.50, CS: 2.50, BA: 2.39
	10 y	**χ^2^** = 6.00, *p* = 0.11, ns.	TV: 2.60, CS: 2.50, CU: 2.45, BA: 2.44
Close/Up views	5 y	**χ^2^** = 11.38, *p* < 0.01	TV: 3.05, CS: 2.53, BA: 2.52, RS: 1.89
	6 y	**χ^2^** = 11.34, *p* < 0.02	TV: 2.86, CS: 2.67, RS: 2.31, BA: 2.15
	8 y	**χ^2^** = 4.89, *p* = 0.18, ns.	TV: 2.69, CS: 2.61, BA: 2.50, RS: 2.19
	10 y	**χ^2^** = 22.45, *p* < 0.0001	TV: 2.88, CS: 2.54, RS: 2.28, BA: 2.28
Side/Top views	5 y	**χ^2^** = 2.57, *p* = 0.46, ns.	CU: 2.73, BA: 2.67, CS: 2.41, RS: 2.18
	6 y	**χ^2^** = 10.81, *p* < 0.02	CU: 3.03, CS: 2.45, BA: 2.37, RS: 2.14
	8 y	**χ^2^** = 33.75, *p* < 0.0001	CU: 3.66, CS: 2.30, RS: 2.05, BA: 1.97
	10 y	**χ^2^** = 24.42, *p* < 0.0001	CU: 3.05, CS: 2.59, BA: 2.27, RS: 2.08
Before/After	5 y	**χ^2^** = 28.27, *p* < 0.0001	CS: 3.58, TV: 2.82, CU: 1.97, RS: 1.62
	6 y	**χ^2^** = 34.29, *p* < 0.0001	CS: 3.84, TV: 2.34, RS: 2.12, CU: 2.04
	8 y	**χ^2^** = 27.29, *p* < 0.0001	CS: 3.61, TV: 2.41, CU: 2.22, RS: 1.75
	10 y	**χ^2^** = 78.88, *p* < 0.00001	CS: 3.81, TV: 2.30, TS: 1.94, CU: 1.93

Table 2 and the associated results showed a dramatic decrease with rising age in the extent to which distractors were chosen by participants (with a corresponding increase in correct answers). Table 4 and the non-parametric Friedman ANOVAs reveal that for most conventions and all age levels, there was also a significant order effect in the extent of distractor choice and relatively high level of stability in those choices. However, for some conventions, (e.g., the realistic-schematic convention) there were no significant order effects in distractor choice except for 5 years old.

### Answer Justification Analysis

As described in the section “Materials and Methods,” verbal justifications were coded according to four categories. Justification categories were (i) *Appropriate*, (ii) *Partially Appropriate*, (iii) *Inappropriate*, and (iv) *None*. Table 5, shows how verbatim examples of typical justifications given by children in each age group were coded into these categories. The coding of these examples was performed by two independent raters using a sample of 25% of the data (the rare discrepancies were resolved by discussion between the raters).

**TABLE 5:** Coded examples of children justifications for each convention.

Justification type Age		Whole/Cross- section Criteria 1. Middle section 2. Internal view	Realistic/ Schematic Criteria 1. Same shape view 2. Stylized	Whole/close Up Criteria 1. Close-up, 2. Bigger-partial	Side view/top view Criteria 1. Orientation 2. Shape	Before/After Criteria 1. After time, 2. State change
Examples of Appropriate justification	5 y	“you can see it’s -*pointing-* cut in half and then again” “here-*pointing-* It’s broken in half and now here it’s broken in half too.”	“here -*pointing-* there it is the same shape and there it is the same shape”	“there -*pointing-* you can see the flower up close and the shoe up close”	“there -*pointing-* you can see the top of the hat and there the top of the egg” “you see the top of the dish and then -*pointing-* you see the top of it, the kiwi”	“we take a banana and then we peel it, we turn it around” “the orange you see it peeled and then here -*pointing-* it’s peeled too”
	6 y	“because the egg is cut in half, so I cut it in half”	“because it’s the same image but in black and white”	“because there -*pointing-* we see it normally and there we see it more closely”	“because the egg is seen from above and the kiwi is seen from above”	“because the orange is peeled and so the banana is peeled” “before there was something around- *pointing-* and now it’s gone and so the egg was cut, so there’s something (less)”
	8 y	“the hat is cut in half and the flower too”	“because the hat is drawn and here-*pointing-* too”	“we see the banana up close and the orange too” “we see the kiwi up close and the flower up close”	“we see the kiwi from above, like the egg from above”	“we take off the headband from the hat and here-*pointing-* we take off the orange peel.”
	10 y	“the cup is cut in half” “the banana is cut there-*pointing-*, the orange is cut there” “because the dish is cut in half and now -*pointing*- it’s the same”	“the shoe is drawn there-*pointing-* so the egg is drawn there”	“there -*pointing-* it is zoomed in.” “there -*pointing-* it’s zoomed in and there too”	“you can see the orange from above”	“because there-*pointing-* we remove the laces and there we remove the petals” “It’s peeled”
Examples of Partially Appropriate justification One criteria, incomplete justification	5 y	“you can see half the egg and half the orange”	“here-*pointing-* the food, the orange, it is white and there the food, the banana, it is white”	“this egg it had become bigger”	“because there -*pointing-* we see what’s at the top and there we see what’s at the top”	“there-*pointing-* it’s cut and then there-*pointing-* it’s cut”
	6 y	“because we can see inside and there-*pointing-* too” “you can see half of it and then again” “it’s because there’s something in it, I think. because the kiwi is cut”	Criteria “the kiwi with colors and there is no color” “because there’s something in the bowl and there’s something to hold the egg.”	“because there-*pointing-* we see correctly and there-*pointing-* we see bigger ones”	“the orange-*pointing-* it is open and the egg too”	“because the skin is torn off” “because now- *pointing*- it’s straight and now you can see it from above”
	8 y	“there’s -*pointing* - half the cup and there’s half the bottle too” “you can see half the orange and half the banana” “the bowl is cut and the kiwi is cut” “the dish is only half full and the kiwi is cut”	“There’s - *pointing-* a drawing.” “it’s a drawing”	“because it’s closer” “we see that part of the cup is bigger and there - *pointing-* we see only part of the banana but bigger”	“because it is seen from top” “here we see the banana lying down and there we see it in height and there -*pointing*- we see the flower lying down and there in height”	“the banana skin is cut and the kiwi is cut”
	10 y	“the banana is cut there; the orange is cut there too” “you can see the inside and there too”	“Here it is in black and white and here too it is in black and white”	“the banana you see in full screen and then the orange too”	“we see her a little high up and then again I think”	“fully open”
Examples of Inappropriate justification Irrelevant or general (global) criteria	5 y	“Here’s -*pointing-* a shoe and here’s a hat.” “you can see that there is still the skin and here -*pointing-* there is still the shell” “This is the kiwi and a half and this is the avocado and a half”	“we see the side, the side, and here - *pointing-* the side, the side and the side” “Now it’s not broken and here - pointing- now it’s not broken.” “we see that the kiwi is ready, we haven’t peeled its skin and here there are no petals that are removed”	“there -*pointing-* you see a round and unpeeled rose and there you see a round and unpeeled egg and the shell remains” “there - pointing- we see it in its entirety and there too”	“when there is wind the petals are removed and the stuff from the flowers is put on the ground” “there’s a kiwi and there’s also food” “this -*pointing*- is big and here this is big” “now it’s the same, you see the whole cup and then you see the whole cup3” “because there- *pointing-* we see the side of the banana and there we see the side of the object”	“here -pointing- the whole shoe and here -*pointing*- this is the half picture”
	6 y	“because now you see a cup on the side” “because there it is whole and there we see it whole too”	“there, -*pointing-* we see correctly and there we see from above”	“there - *pointing-* you have to find half of it.”	“because there -pointing- it is whole and there it is also whole” “there, - pointing- the hat is fine and there the kiwi is broken”	“because you can see the inside of the bowl and then you can see the top of the orange”
	8 y	“we see the flower in profile and the cup too”	“the two are not too distant” “it was empty and the cake pan was empty”	“because it’s cut off and here -pointing- too” “because you can see it from behind”	“because it’s different.” “because it’s closer.” “here we could see the cup and the inside of the cup and there we can see the inside of the shoe”	“the hat is a little torn and there, the shoe too” “in the dish there is a cake and in the egg there is the egg white”
	10 y	“this one -pointing- removed petals and there’s a little orange juice” “he is lying down”	“Because it is cut here - pointing-, and here too it is cut”	“Both they’re a little… how to explain, they’re in the way.”	“Here, -pointing it’s closer. and here too it’s closer” “It is seen closer”	“he just lost something and here too- pointing-”

*Each example is a verbatim of the spoken justification of the child. We have added the word *pointing* to justifications when the child was pointing to, or otherwise indicating an item. One or two typical examples of each justification category are reported for each convention and age.*

A number of observations can be made from the qualitative data reported in Table 5 on how much children were engaged in the task, trying to actively and cleverly, sometimes with huge creativity, interpret conventions meaning from the analogy task. More specifically, (i) For a given convention, language use (words, nouns, adjectives, verbs, prepositions) in the justifications tended to change considerably with increasing age. For example, for the side/top view convention, only older children used the following type of description: “we see the kiwi *from above*, like the egg *from above*”; whereas the younger children more often used descriptions like: “there you can see *the top of* the hat and there *the top of* the egg”; (ii) Older children tend to mention both criteria (see Table 1) for each convention more often than did younger children. (iii). Some words used to describe a convention are produced only by older children, because younger children lack this *“technical”* vocabulary to describe the convention (for example, to describe the realistic/schematic convention older children, 8–10 years old) used the expression “the shoe is *drawn* there so the egg is *drawn* there”). However, younger children may nevertheless answer correctly, despite not being able to produce the most relevant vocabulary in their justifications. This question will be one of the issues to be considered later in this section where quantitative analysis of the answer justifications is reported in relation with hypothesis 1 (Table 6).

**TABLE 6:** Mean percent (and SD) of each category of justification, at each age and for each convention type.

Justification type Age		Whole/Cross- section	Realistic/Schematic	Whole/close up view	Side view/top view	Before/After	Mean
Appropriate	5 y	56.78 (31.55)	30.72 (35.25)	47.71 (40.21)	12.41 (24.49)	37.25 (21.85)	37.14 (21.00)
	6 y	55.25 (27.71)	53.82 (43.01)	78.12 (22.84)	36.11 (27.73)	47.57 (18.78)	58.49 (22.85)
	8 y	76.54 (22.18)	80.24 (32.23)	87.65 (12.14)	64.19 (22.72)	54.94 (18.06)	71.48 (20.85)
	10 y	83.33 (25.67)	79.82 (37.86)	87.72 (24.61)	68.71 (26.70)	67.25 (23.26)	71.48 (29.99)
Partially appropriate	5 y	8.50 (10.78)	5.88 (16.25)	9.80 (16.14)	12.42 (17.95)	20.26 (19.73)	11.65 (10.37)
	6 y	10.76 (15.32)	21.18 (36.29)	1.74 (4.10)	7.98 (9.46)	9.03 (11.08)	4.08 (4.40)
	8 y	4.32 (6.75)	14.81 (29.27)	1.23 (3.59)	7.41 (9.33)	6.17 (7.83)	6.79 (11.35)
	10 y	0.87 (3.98)	9.35 (27.15)	0.88 (3.03)	6.72 (12.5)	8.18 (12.09)	5.80 (4.64)
Inappropriate	5 y	14.38 (17.90)	35.29 (33.84)	24.18 (28.66)	44.44 (31.67)	20.26 (16.78)	28.15 (19.37)
	6 y	23.61 (19.30)	20.14 (32.26)	10.76 (9.56)	38.88 (30.52)	31.94 (14.87)	23.64 (12.91)
	8 y	17.90 (20.57)	4.32 (9.44)	9.87 (13.14)	21.60 (15.93)	32.71 (18.07)	17.28 (15.43)
	10 y	8.18 (11.75)	0.87 (3.04)	3.22 (7.26)	12.28 (14.79)	16.08 (12.84)	6.42 (7.14)
None	5 y	19.60 (25.01)	27.45 (34.05)	16.99 (27.25)	30.72 (30.81)	19.61 (26.21)	20.45 (25.54)
	6 y	11.45 (17.73)	4.51 (12.89)	8.68 (19.90)	14.58 (22.47)	9.37 (15.99)	13.03 (17.95)
	8 y	0.62 (2.62)	0.62 (2.61)	1.85 (4.26)	5.55 (8.73)	4.94 (5.68)	2.71 (4.78)
	10 y	7.60 (22.24)	9.94 (29.54)	7.61 (23.83)	9.64 (22.54)	7.30 (18.31)	13.49 (25.56)

Justification quantitative data were analyzed with repeated measures MANOVA and non-parametric Kruskal–Wallis ANOVAs, which were performed for each category of justification, including age group as the between subject factor and convention type as the within subject factor.

For the *appropriate* justification category, the analysis revealed an increase in appropriate justifications with age, *F*(3, 101) = 19.93, *p* < 0.000001, ηp^2^ = 0.37, an effect of the convention type *F*(4, 404) = 28.01, *p* < 0.000001, ηp^2^ = 0.22, and a significant interaction between age and convention type, *F*(12, 404) = 3.05, *p* = 0.0004, ηp^2^ = 0.008. The increase in appropriate justification with age did not follow the same pattern, for all conventions. As shown in Table 5, the differences between conventions tended to be higher for 5 years old than for the 10 years old.

Kruskal–Wallis ANOVA supported this result: (i) For the whole-cross section convention, *H*(3, 105) = 25.31, *p* < 0.00001 (mean ranks for 5,6,8, and 10 years old respectively, 40.52, 36.50, 58.22, 70.00) (ii) For Whole/close-up view convention *H*(3, 105) = 19.34, *p* = 0.0002 (mean ranks, 21.44, 46.14, 57.25, 66.40) (iii) For the side-top views convention, *H*(3,105) = 41.98, *p* < 0.00001, (mean ranks, 20.35, 41.29, 66.17, 71.22), (iv) For the realistic-schematic convention, *H*(3, 105) = 19.79, *p* = 0.0002 -mean ranks, 30.79, 46.18, 62.55, 64.06) and (v) For the before-after convention, *H*(3, 105) = 29.30, *p* < 0.00001 (mean ranks, 30.55, 42.31, 52.27, 72.38).

Conversely, for the *partially appropriate* justification category, there was a significant decrease with age, *F*(3, 101) = 3.05, *p* = 0.032, ηp^2^ = 0.08, an effect of the convention type, *F*(4, 404) = 5.37, *p* < 0.001, ηp^2^ = 0.05, and a significant interaction between convention type and age (*F*(12, 404) = 1.98, *p* = 0.024, ηp^2^ = 0.055). Table 5 shows a particularly dramatic drop between 5–6 years old and 8–10 years old, which corresponds (in French schools) at the end of the kindergarten time (6 years old) and the beginning of primary school (7 years old).

Again, the Kruskal–Wallis ANOVA partially confirmed these results (i) For the whole-cross section convention, *H*(3, 105) = 17.08, *p* = 0.0007 (mean ranks respectively for 5,6,8 and 10 years old, 62.38, 62.26, 53.66, 40.68) (ii) For Whole-close-up view convention *H*(3, 105) = 8.79, *p* = 0.032 (mean ranks, 65.00, 52.81, 50.55, 48.94) (iii) For the side-top views convention, *H*(3, 105) = 1.84, *p* = 0.60, (mean ranks, 56.64, 55.79, 54.52, 48.28), (iv) For the realistic-schematic convention, *H*(3, 105) = 4.97, *p* = 0.17 (mean ranks, 50.73, 58.71, 56.97, 47.31), and (v) But, for the before-after convention *H*(3, 105) = 7.27, *p* = 0.63, ns. (mean ranks, 69.20, 52.39, 47.33, 48.94).

For the *inappropriate justifications*, repeated measures MANOVA revealed a decrease with age, *F*(3, 101) = 16.62, *p* < 0.00001, ηp^2^ = 0.33; an effect of the convention type, *F*(4, 404) = 17.41, *p* < 0.00001, ηp^2^ = 0.15; and a significant interaction between age and convention type, *F*(12, 404) = 4.70, *p* < 0.00001, ηp^2^ = 0.12. Finally, for the *no-justification* category, repeated measures MANOVA showed a decrease with age (*F*(3, 101) = 3.60, *p* = 0.016, ηp^2^ = 0.09), and the absence of justification was proportionally higher for difficult conventions (side-view/top-view) than for simpler conventions (whole/close-up; realistic/abstract), *F*(4, 404) = 5.37, *p* = 0.0003, ηp^2^ = 0.05. This finding suggests that some types of convention are far more difficult for young children to explain than others. The interaction between age and convention type was also significant, *F*(12, 404) = 2.32, *p* = 0.007, ηp^2^ = 0.06.

**FIGURE 4:**
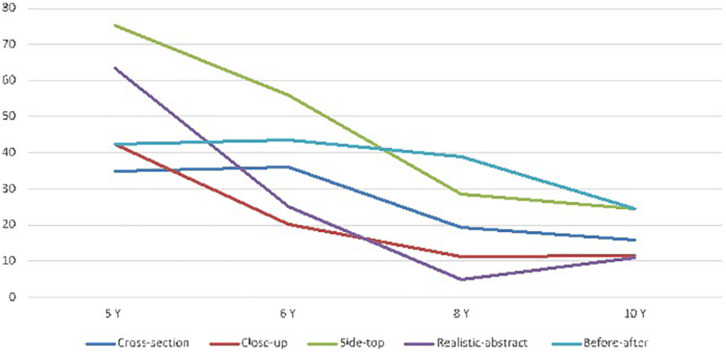
Mean proportion (%) of inappropriate and no-justifications at each age group and convention.

The two significant interactions between inappropriate justifications or no-justifications and convention types were analyzed in more detail using Kruskal–Wallis ANOVAs for each convention on inappropriate and no-justifications in combination. (i) For the whole-cross section convention there was a significant and progressive decrease of inappropriate and no-justifications, starting at around 8 years of age, *H*(3,105) = 18.05, *p* = 0.0004 (mean ranks respectively for 5,6,8 and 10 years old: 64.20, 66.75, 47.36, 39.07). (ii) For the whole-close-up convention, a similar but more dramatic progressive decrease of inappropriate and no-justifications was found, *H*(3,105) = 18.81, *p* = 0.0003 (mean ranks respectively for 5,6,8 and 10 years old: 72.76, 60.73, 49.44, and 39.32). (iii) For the side-top convention the decrease tended to occur from the oldest children group, *H*(3,105) = 36.88, *p* < 0.00001 (mean ranks respectively for 5,6,8 and 10 years old: 81.76, 65.53, 41.80, 34.89). (iv) For the realistic-abstract convention the decrease started earlier in the 6 years old group, *H*(3,105) = 29.92, *p* < 0.00001 (mean ranks respectively for 5,6,8 and 10 years old: 82.73, 54.82, 43.15, 42.44). Finally, for the before-after convention, the stronger decrease occurred in the oldest children group, *H*(3,105) = 20.36, *p* = 0.0001 (mean ranks respectively for 5,6,8 and 10 years old: 61.85, 65.82, 58.36, 35.69). These tendencies are summarized using the data from Figure 4.

Further insights into the relation between the analogy task performance and the corresponding justifications can be obtained from an analysis of the degree of fit between participant choices in the analogy task and how they were justified. In principle, correct choices should be accompanied by justifications that are consistent (rather than inconsistent) with those choices. Consequently, there should be high positive correlations between correct choices and appropriate justifications but negative correlations with inappropriate justifications. To examine this issue, appropriate and partially appropriate justifications were combined into one group and their correlations with correct choices for each of the conventions compared with those of the incorrect justifications. The results given in Table 7 and show the expected pattern of correlations. Although the choices were not always properly justified, the correlations indicate that correct choices were mostly reasoned rather than a result of chance.

**TABLE 7:** Correlations (Bravais-Pearson *r*) between good answers and justifications, good+close and wrong (*p* < 0.001, for all the values of the table).

Good Answers	Whole/Cross- section	Realistic/Schematic	Whole/close up view	Side view/top view	Before/After	Total
Good Justifications	0.73	0.48	0.76	0.67	0.72	0.75
Good + close Justifications	0.69	0.62	0.71	0.64	0.58	0.65
Wrong justifications	−0.74	−0.68	−0.75	−0.47	−0.52	−0.74

Finally, a closer inspection of Table 2 showing the answers for each convention on the analogy task, and of Table 6, showing the percent of appropriate, partially appropriate and non-appropriate justification, revealed a numerical difference between the mean percentage of correct answer for the analogy task and the mean percentage of appropriate and partially appropriate justifications. This difference was calculated, for each convention with the results presented in Table 8.

**TABLE 8:** Mean differences (in %) between the analogy task performance scores and the justification scores, for each convention, for two levels of appropriateness of the justification (respectively for the fully appropriate justifications only and for the fully plus partially appropriate justifications) at each age group.

Performance minus justification	Age	Whole/Cross- section	Realistic/Schematic	Whole/close up view	Side view/top view	Before/After
Appropriate Justifications only	5 y	+ 2.12(22.74)	+ 40.93(36.07)	+ 17.32(23.72)	+ 40.52(29.64)	+ 8.78(14.06)
	6 y	+ 13.80(22.86)	+ 35.24(38.45)	+ 6.55(15.88)	+ 21.74(21.39)	+ 11.47(15.82)
	8 y	+ 4.94(7.83)	+ 17.20(30.94)	+ 3.08(5.12)	+ 16.66(19.52)	+ 10.95(9.16)
	10 y	+ 2.34(19.53)	+ 19.01(37.15)	+ 5.84(22.48)	+ 12.27(22.93)	+ 6.06(16.98)
Appropriate + partially appropriate Justifications	5 y	−6.37(24.42)	+ 35.05(38.86)	+ 7.52(22.98)	+ 28.10(31.94)	−11.47(23.16)
	6 y	+ 3.04(20.64)	+ 14.06(27.28)	+ 4.81(16.18)	+ 13.75(25.35)	−2.44(14.85)
	8 y	+ 0.61(19.53)	+ 2.39(8.70)	+ 1.85(4.26)	+ 9.25(17.97)	+ 4.78(7.96)
	10 y	+ 1.46(8.91)	+ 9.65(28.78)	+ 4.97(22.77)	+ 5.55(21.88)	−2.11(19.87)

*A + sign means that performance on the analogy task was higher than the justification performance. A − sign means the reverse.*

Table 8 revealed a major trend: answer performance scores are mostly higher than the justification scores. This is always true for the fully appropriate justification level and also, to a lesser extent, for the fully appropriate plus partially appropriate justification level. Given that finding the correct answer by chance among five choices (e.g., 20%, among a series of 5 items including 4 distractors which are highly related), is relatively unlikely, this trend may indicate that children understood the convention but still had insufficiently developed language capacities to explain their understanding completely.

Further, such language and verbalization difficulties seemed to be higher for some conventions than for others (for example for the realistic-schematic and for the side view -top view conventions, performance on the analogy task appear much higher than the ability to justify the task answer verbally). As already mentioned above, the before after convention seemed to have a different “status” than the others. It could well be that the before-after convention mainly provided learners with a general temporal feature which is in fact shared with other convention (such as the whole-cross-section, the realistic-schematic or the whole-close-up view). This general aspect of the before-after convention may explain why it was frequently conflated with other conventions.

Finally, Table 7 indicated also age group differences. In the 5 years old age group, and for two conventions (the whole-cross section and the before after conventions) several children generated a partially appropriate justification whereas the answer selection was incorrect. This might be due to the fact that some conventions could share one common general feature (such as, for example, the temporal feature and/or a superficial perceptual common feature). However, this mismatch never happened for the fully appropriate justification level which included two criteria. In sum, the analogy task answers seem to be a more reliable measure of the “actual” level of understanding of the conventions than the justification themselves.

### “Implicit” Learning Effect Possibility?

In the present study, 5 types of different conventions were tested with a series of items presented for each convention type in a within-subjects’ experimental design. Given the potential of analogical learning exercises to improve relational abstraction (Kurtz et al., 2013; Stevenson et al., 2013, 2014; Thibaut and Goldwater, 2017), this possibility should be considered for the present study by examining if performance changed across the 45 trials. Such measure would be relatively novel because it contrasts with previous studies that mainly employed dynamic testing which included feedback. However, the result of this “potential learning effect” analysis should be viewed with caution in the present case because (i) the 45 items were delivered randomly and so ordered differently for each subject, (ii) as shown above, the conventions differed in difficulty (for example the realistic-schematic convention was easier than the side-top view convention), and (iii) for each convention, there were within category items and between category items, this feature adding a variation in the semantic distance. In sum, the trials were of unequal difficulty, with different random position in the row of the 45 trials across subjects. These experimental constraints could pose severe limitations on the interpretation of the results of this learning effect analysis.

**FIGURE 5:**
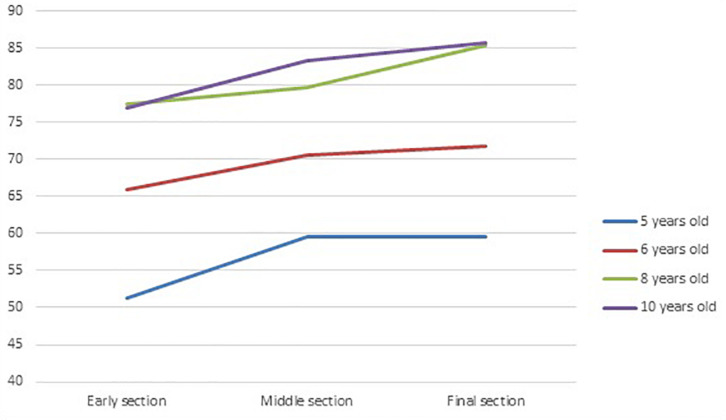
Percentage of good answers according to age groups and items sections.

In order to investigate whether the number of correct answers changed across time, the 45 trials were divided into three sections comprising respectively for the first, early section the eleventh first presented items, for the third, final section, the eleventh last presented items, and for the second middle section the 23 items that were presented in the middle of the row. There was a rationale for making such a subdivision of the items. The objective was to compare a small set of starting elements to a similar small set of final elements, separated by a larger set of elements during the resolution of which a potential learning effect may occur, but this choice of subdivision can of course be contested. The percent of correct answers for each section was then calculated for each age group. Results are presented in Figure 5.

A repeated measures ANOVA was performed on the percentage of correct answers, with age groups as the between subjects’ factor and the three items sections as the within subjects’ factor. As shown above, a strong improvement of the percentage of good answers according to age group was found *F*(3, 101) = 21.22, *p* < 0.00001, ηp^2^ = 0.36. A significant effect of the section was found with an increase of good answers from the early section of items to the final section, *F*(2, 202) = 13.16, *p* < 0.00001, ηp^2^ = 0.11. Univariate comparisons indicated significant differences between the early section and the middle section (*F*(1,101) = 11.24, *p* = 0.001), the early section and the final section (*F*(1,101) = 22.52, *p* < 0.00001); but not between the middle and the final sections (*F*(1,101) = 2.78, *p* = 0.098, ηp^2^ = 0.01). Further there was no significant interaction between age group and sections, *F*(6, 202) = 0.45, *p* = 0.84). Although all age groups seemed to have learnt across trials, the extent of this learning effect was comparatively modest at from 8 to 10 percent.

## Discussion and Conclusion

This study investigated the development of comprehension of paired graphics conventions in children. Paired graphics depicting everyday subject matter familiar to children were devised to instantiate five widely used graphic conventions: normal and close-up views; before and after views; whole and cross sectional views; realistic and abstract depictions; side and top views. An analogy task based on these paired graphics was developed to assess how well these five conventions were understood by children aged 5, 6, 8 and 10 years.

For the five conventions included in this study, comprehension level increased with age. Further, at each age there were differences in the extent to which the individual conventions were understood. This finding is new and has never been shown before empirically and experimentally. In no case did 5 years old reach the *a priori* threshold of 75% correct (which is conventionally often used in psychometrics measures, Lord and Novick, 1968; Cohen, 1977; Nunnally and Bernstein, 2010; Gescheider, 2015) we considered a reasonable criterion for satisfactory understanding. This is consistent with their few exposures to graphic conventions but may also reflect their general level of cognitive development. Once children were in their 1st year of schooling, some scores (realistic/abstract; normal/close-up) exceeded the comprehension criterion threshold. However, there was little difference in 5 and 6 years old scores for the remaining conventions (whole/cross-section, side view/top view, and before/after). In contrast, 8 years old children (2nd year of the primary school) scores reached 75% for almost all conventions. Further, scores are still rising in the 10 years old children who had scores considerably above the 75% threshold. Taken together, these results suggest an age-related development in the capacity to understand usual graphics conventions and to make progressively finer discriminations between the various conventions that are used in paired graphics, but also that some conventions remain more problematic than others.

Further, our analogy task appeared to be a more reliable measure of the “actual” level of understanding of the conventions by children than the justification task which may have been constrained by language and verbal explanation difficulties encountered by the young children. Importantly, our results indicated also that children (especially the oldest) were able to generalize the meaning of the conventions from prototypes exemplar (a cross section of an orange) to an unfamiliar exemplar (a cross section of a hat): this shows that the conventions meaning became more abstract, like a more general “rule.”

Our results revealed also that younger children were actively engaged in trying to interpret and find the meaning of the conventions, using all potential cues given by or rising from the comparison process of the pairs of pictures. Finally, objects knowledge names was controlled in this experiment. To sum up, the results demonstrated that most participants had developed understandings of graphical conventions by age 10, presumably as a function of incidental exposure to those conventions in textbooks, and electronic educational support. So it could be expected that an increase in exposure to graphics may lead students to learn conventions more quickly. Our results suggest that pupils (and teachers) should engage with diagrammatic and graphical content more intentionally.

Furthermore, the results seem to offer more detailed information about the timing, design, and use of these graphical conventions across young children’s schooling experiences.

Even if there were correct and incorrect answers, with a clear increase of correct answers with age, when the younger children gave an incorrect answer, they often chose answers that could be considered, if not correct, as “valid” and not totally invalid or random. For example, choosing the before/after convention instead of the whole/cross section convention is not an absolute wrong answer, because both conventions share a temporal feature. However, our results indicated also that children acquired a more precise and specific meaning of the conventions. It must be acknowledged, however, that the before/after convention, although very common in primary school textbooks, appears different (in nature) from the four others.

Further, interestingly, during the time on the analogy task, even the youngest children were attending to relationships between the pictures (for example from Table 4, we can see that some children noted that certain pairs had *skins* other not). This fundamental ability to comparison seems very early. However, our results suggest also another developmental trend: younger children more often based their comparison activities on perceptual features of the pictures, while older children based their answers on more general features or “rule,” e.g., specific and more abstract meaning of the convention. This finding appears to be particularly consistent with the model of “relational shift” developed by Gentner (1988) and confirmed in Rattermann and Gentner (1998). The relational shift hypothesis (RSH) proposes that children interpret analogy and metaphor first in terms of object similarity and then in terms of relational similarity. Gentner & al. research showed mainly that in analogy tasks, (i) object-similarity errors were highly frequent initially in young children (4 years old) and decreased with age; (ii) the rate of relational (correct) answers increased with age; and (3) performance on the analogs was positively related to children’s knowledge about the participating causal relations. Our trend of result could be an indication for text book graphic designers, to use for example cueing techniques which signal and direct learners attention on the conceptually relevant features but not on the perceptually salient but less relevant features (see de Koning et al., 2007, 2010a, 2010b; Boucheix and Lowe, 2010; Boucheix et al., 2013b).

However, despite these results suggesting the possibility of age-related development in the capacity to understand usual graphics conventions, this initial experimental study of such capacity development has a number of limitations, particularly with regard to its scope.

(i) Paired-graphics used in this study did not include neither explanatory text nor scaffolding techniques as a school teacher would sometimes do in a more ecological situation. According the multimedia principle (see Mayer, 2014), the adding of verbal, aural or textual information, captions and other additional textual or graphic information to the paired graphics, in schoolbooks, may enhance and increase comprehension and learning. However, text-picture integration activities required in such multimedia presentations may increase cognitive demand and cognitive load. However, follow-up studies including a scaffolding condition might be most illuminating. Further, our material used known objects, which did not require prior knowledge, and despite the absence of explanatory text or captions accompanying the pairs, which was intended for methodological and scientific reasons, children well understood the task and its expectation. Would it be possible that some of the lower performance of young children would be mitigated if they had more context and text accompanying the paired pictures, or are encountering these as part of a designed instructional sequence? This issue could be the goal of future studies. However, at first sight, this assumption is not so likely, given the actual “poor” or at least unprincipled design of the accompanying texts in school textbooks (see Boucheix et al., 2013a). As suggested by these authors, in their empirical investigation of primary school text-books comprehension, text and context seem to be often suboptimal and the learners should deal with inconsistency between graphics and their textual context. There could be a misalignment between what textbook designers are realizing and what is more comfortable, better suited, for early aged pupils in terms of context, transparency of the verbal explanations accompanying the graphics and also relatively to the presence of referential connections between text and pictures (Désiron et al., 2018). The present results may provide useful information about age related ability to understand graphics conventions. During the implicit and progressive acquisition of conventions, meaning may arise in response to “a need,” so it could also be another issue to look at the intersection of task, student, and task expectation (Di Sessa, 2004). But this issue appears more difficult to investigate experimentally. The implications for teaching graphicacy may be a call to engage in multimodal literacy to study if and how teachers scaffold graphics comprehension, and to examine comprehension of graphics in better text-book design.

(ii) In the present study, the design of the analogy task items seemed to be quite challenging for young children because each item included five available choices (one good answer and four distractors), with all being somewhat related with each other. It could be interesting, as a follow up to the present study, to narrow the number of distractors to just the correct option and the most prototypical, frequent or popular distractor. In the same set of ideas, perceptual features of the distractors could be manipulated (for example, perceptually salient but conceptually irrelevant). Similarly, in the present study the semantic distance (within entity category vs. between entity category) between the objects of the base pair and the objects of the target pairs was controlled. For example, it was expected that it would be easier to correctly identify a cross-section of an orange if the base pair depicted a kiwi fruit than if the base pair depicted a hat. Such an items analysis was not in the scope of this study, but the results suggested that semantic distance had an effect and especially within category convention items were easier than between category convention items.

(iii) It could also be interesting to explore the effects of explicit comparison, either between examples of the same convention or examples of different types. This idea of explicit comparison during multiple graphics processing might also be connected to scaffolding technique which could be used by teachers in order to help students to build convention meaning.

(iv) Previous research showed that preschool children are able to detect an abstract relation (and override object matches) when they explicitly compare two examples of the relation (e.g., Christie and Gentner, 2010), so they may show sensitivity to the graphic conventions with this added instruction.

(v) In the present research we found that performance changed and significantly improved across the 45 trials. This improvement, although significant, has been modest. However, this result must be taken with caution, due to the unstructured random presentation of the items and to their unequal difficulty. It may well be that exposure to analogies, during sequences of items presented in a progressive and structured manner, will have a greater impact on learning, for example including a progressive abstraction, as in the study by Thibaut and Goldwater (2017). Moreover, these sequences could eventually be accompanied by a scaffolding of comparison activities. This issues would be worth addressing in follow-up studies.

Finally, this research may have implications for the design and use of instructional images, such as graphic conventions. Regarding designers, firstly the necessary better (optimal) alignment between perceptual salience and thematic reliance of graphic (Lowe, 1999) should be rethought in the light of graphics (static as well as dynamic) cognitive processing constraints (Lowe and Schnotz, 2008). Secondly, graphic conventions are not transparent objects that could be “naturally” easily interpreted. As a consequence, sometimes adds-on or ancillary information such as signaling or cueing techniques cueing could be used. In addition, the “coherence” (e.g., the coherence principle in Mayer, 2014) between text and picture should be of better quality (Richter et al., 2016). Regarding the acquisition of convention and more generally of graphicacy, the use of instructional images should be more principled. Our results suggest that teachers may be more engaged in graphic convention learning. The development of the understanding of graphics convention may require more scaffolding. The use of comparison tasks, of progressive complexity, (such as analogy task) as a learning tool may well be tested.

In conclusion, as yet, there is little empirically based evidence available to guide curriculum developers who may be charged with addressing the present lack of “graphicacy” tuition in schools. Graphicacy is a multi-faceted capacity so the study reported here is necessarily limited because it was restricted to paired graphics and only a subset of the conventions used in this form of depiction (Wilmot, 1999; Aldrich and Sheppard, 2000; De Vries and Lowe, 2010). Further, the focus of the present investigation was on broad developmental issues rather than more detailed matters such as the perceptual and cognitive processes that learners engage in when dealing with paired graphics. Methodologies such as eye-tracking could help to explore these and other processing issues. Of particular interest are the extent to which learners engage in comparisons between the two pictures comprising a graphic pair, the nature of those comparisons, and the relationships between intra-picture and inter-picture interrogations.

## Data Availability Statement

The original contributions presented in the study are included in the article/supplementary material, further inquiries can be directed to the corresponding author.

## Ethics Statement

The studies involving human participants were reviewed and approved by official agreement between the Academia Inspection of the French National Education Ministry and the University. Written informed consent to participate in this study was provided by the participants’ legal guardian/next of kin.

## Author Contributions

J-MB made substantial contributions to the conception or design of the work, acquisition, analysis and interpretation of data, and wrote the draft the work. RL contributed to the conception of the work, English language revision, and critical revision of the draft for important intellectual content. J-PT contributed to conception of the analogy task and revised the draft critically for important intellectual content. All authors contributed to the article and approved the submitted version.

## Conflict of Interest

The authors declare that the research was conducted in the absence of any commercial or financial relationships that could be construed as a potential conflict of interest.
